# Evaluation of medication errors in patients with kidney diseases in Quetta, Pakistan

**DOI:** 10.1371/journal.pone.0289148

**Published:** 2023-08-02

**Authors:** Tahira Bano, Noman Haq, Aqeel Nasim, Muhammad Saood, Maria Tahir, Riffat Yasmin, Nisar Ahmed, Ghulam Razzaq, Shabana Qudos, Abdul Kareem Zarkoon, Muhammad Shafi

**Affiliations:** 1 Faculty of Pharmacy and Health Sciences, University of Balochistan, Quetta, Pakistan; 2 Provincial Drug Testing Laboratory Balochitan, Quetta, Pakistan; 3 Sardar Bahadur Khan Women University Quetta, Quetta, Pakistan; 4 Balochistan Institute of Nephro-Urology Quetta, Quetta, Pakistan; 5 BMC College Quetta, Quetta, Pakistan; University of Gondar, ETHIOPIA

## Abstract

**Background:**

Medication errors represent a significant challenge in healthcare, as they can lead to enduring harm for patients and impose substantial financial burdens on the healthcare system. To effectively mitigate medication errors, it is imperative to gain a comprehensive understanding of their frequency and the contributing variables. Thus, the primary objective of this study was to evaluate the occurrence of medication errors among patients with kidney diseases in Quetta, Pakistan.

**Methods:**

The objective of this study was to assess medication errors in patients diagnosed with kidney diseases in Quetta, Pakistan. The research was conducted at the Balochistan Institute of Nephro-Urology Quetta (BINUQ) Hospital, which serves as a tertiary care center specializing in the treatment of kidney diseases. A cross-sectional descriptive study design was employed over a period of six months. The study population consisted of patients admitted to the Nephro-urology wards at BINUQ Hospital during the specified duration. Data collection encompassed various methodologies, including checklist-guided observation, review of prescription order forms, documentation of drug administration, and comprehensive analysis of patient medical records. Descriptive and analytical analyses were conducted using SPSS version 23. Univariate analysis was employed to identify independent variables associated with medication errors, employing a significance level of p<0.01. The multivariate logistic regression analysis incorporated variables that exhibited a significant association with medication errors during the univariate analysis. Only those variables demonstrating a p-value of less than 0.05 at a 95% confidence level were considered significant predictors of medication administration errors within the final multivariate model.

**Results:**

Among the 274 medication errors identified in the study, documentation errors accounted for 118 cases (12.06%), administration errors for 97 cases (9.91%), prescribing errors for 34 cases (3.47%), and dispensing errors for 25 cases (2.55%). Statistical analysis revealed significant associations (p<0.05) between forgetfulness and duty shift, and medication errors in the documentation process. Similarly, inattention was significantly associated (p<0.05) with both prescribing and dispensing errors. Furthermore, the number of medications received emerged as the most influential factor associated with medication errors. Patients receiving 4–6 medications exhibited an odds ratio of 9.08 (p<0.001) compared to patients receiving 1–3 medications, while patients receiving more than 6 medications had an odds ratio of 4.23 (p<0.001) in relation to patients receiving 1–3 medications.

**Conclusion:**

In conclusion, this study determined that documentation errors were the most prevalent medication errors observed in patients with kidney disease in Quetta, Pakistan. Forgetfulness and duty shift were associated with documentation errors, whereas inattention was linked to prescribing and dispensing errors. The significant risk factor for medication errors was found to be a high number of prescribed medications. Therefore, strategies aimed at reducing medication errors should prioritize enhancements in documentation practices, alleviating medication burden, and increasing awareness among healthcare providers.

## Introduction

Medication errors pose a significant risk to patient safety [1]. These errors, along with adverse events, are major contributors to preventable deaths and healthcare expenditures on a global scale [2]. Among the various types of medical errors, the most common medical error is inappropriate medication administration [3]. Currently, one in ten patients reportedly experiences unintended medical errors. This has led to the World Health Organization classifying this issue as endemic [4].

Medication errors represent a significant public health issue in the United States and are a leading contributor to mortality. Identifying the underlying factors that contribute to these errors and devising efficacious strategies to prevent their recurrence pose formidable challenges [1]. To improve patient safety, it is essential to recognize and learn from incidents and take proactive measures to prevent their reoccurrence [5].

Patient safety is a global problem that has an impact on people’s health [6]. Medication errors are the leading cause of harm in the United States, affecting almost 1.5 million people and costing the healthcare system $3.5 billion each year [7]. The annual global economic impact of medication errors is estimated to be $42 billion [8]. The World Health Organization (WHO) launched a global patient safety program named "Medication without Harm" in March 2017. Within five years, the program seeks to reduce drug-related preventable damage by half in all countries [9, 10].

Several studies have revealed that physician ordering is the stage of integrated medicine delivery most usually related with errors, followed by nursing administration, transcription errors, and pharmacy dispensing errors [11]. The most frequently reported issues include improper medication, incorrect dosage or frequency, incorrect route of administration, failure to recognize drug-drug interactions, inadequate monitoring, missed or delayed doses, and difficulties with communication [12]. Adverse drug events (ADEs) and MEs are prevalent in various clinical settings and can occur at any stage of the medication-use process. MEs are associated with one-third to half of all ADEs [13]. Medication errors contribute to 18.7% to 56% of all ADEs, resulting in negative patient health outcomes and adverse economic effects among hospitalized patients every year [14].

Usually, a doctor prescribes medicine, and a pharmacist dispenses it, but the registered Nurse is responsible for proper medication administration [15]. The Nurse’s role in medication administration may include preparing, checking, and dispensing medications, updating their pharmaceutical knowledge, evaluating therapy outcomes, reporting adverse reactions, and educating patients about their prescriptions [16]. Despite this, several studies suggest this process is not always followed [17].

Errors can happen due to a lack of knowledge, inadequate performance, and psychological issues [1]. Pharmacists play a crucial part in the investigation and development of the healthcare system for patient safety with physicians, nurses, and administrators [18]. The National Coordinating Council for Medication Error Reporting and Prevention (NCCMERP) defines a medication error as an avoidable occurrence that could result in inappropriate medication use or harm to the patient, whether it happens while the medication is under the control of a healthcare provider, patient, or consumer [19].

Although there is a lack of accurate statistical information on medication errors in developing nations, it does not necessarily mean such errors do not occur [1]. These errors can result in prolonged hospital stays, increased costs, and, in some cases, severe injury or even death [20]. These errors can lead to direct harm to the patient, increased healthcare costs, and indirect harm to healthcare workers on a personal and professional level, which can lower their performance and self-esteem [21, 22]. Each year, up to 500,000 people, including women and children, die in Pakistan as a result of medication errors such as incorrect prescriptions, drug overdoses, self-medication, and adverse drug effects [23].

Medication errors are an ongoing issue in healthcare, resulting in adverse events, higher morbidity and mortality rates, and higher healthcare costs [24]. Kidney disease patients are particularly vulnerable to medication errors due to their complex medication regimens, comorbidities, and physiological changes. However, there is a lack of studies on medication errors among kidney patients in Pakistan, particularly in Quetta. This study addresses this gap by evaluating medication errors among kidney patients in Quetta, Pakistan. The findings of this study can provide valuable information on the prevalence, causes, and types of medication errors in this population. This can inform the development of targeted interventions and policies to prevent and reduce medication errors in kidney patients, improving patient safety, better health outcomes, and reducing healthcare costs. This study aims to evaluate the prevalence, causes, and types of medication errors among kidney disease patients in Quetta, Pakistan.

## Methods

### Study design, setting and duration

A cross-sectional descriptive study was conducted six months, from January 2021 to June 2021, at the Balochistan Institute of Nephrology Urology Quetta (BINUQ).

### Study population and sample size

The study included all the registered inpatients of Balochistan Institute of Nephrology and Urology Quetta who were admitted to wards for any treatment.

### Criteria

#### Inclusion criteria

- All those patients were admitted to the BINUQ wards for treatment.
- Both genders.
- Patients aged above 18 years.

#### Exclusion criteria

- Dialysis patients.
- Emergency Department patients.
- Patients aged less than 18 years.

### Study tool

The study tool used for data collection in this study was a checklist. The research team developed this checklist and underwent pilot testing before the start of the study to ensure its reliability and validity. The checklist was utilized to facilitate checklist-guided observations, review prescription order forms, document drug administration, and record various patient medical data.. It included the assessment of medication errors such as omission errors, wrong time errors, unauthorized drug errors, improper dose errors, wrong dosage form errors, incorrect drug preparation errors, incorrect drug administration techniques, deteriorated drug errors, monitoring errors, compliance errors, and other errors. This checklist allowed for a comprehensive assessment of medication errors in patients with kidney diseases in Quetta, Pakistan.

### Data collection

The data was collected by trained research assistants using checklist-guided observation, evaluation of prescription order forms, documentation of drug administration, and other patient medical data. Data was gathered at the inpatient department to assess medication errors such as omission errors, wrong time errors, unauthorized drug errors, improper dose errors, wrong dosage form errors, incorrect drug preparation errors, incorrect drug administration techniques, deteriorated drug errors, monitoring errors, compliance errors, and other errors. The admission and discharge dates were recorded in the inpatient case record review.

All observed errors were recorded and assessed using the NCCMERP index for the following factors: patient age and gender, number of drugs per prescription, length of hospital stay, type of medication errors, and severity of errors. The relationship between age and errors, medication usage and errors, and length of stay with errors was also investigated [25].

Upon detection of medication errors (MEs), an effort was undertaken to pinpoint the factors contributing to these errors. The involvement of diverse healthcare professionals (HCPs) and types of errors, including knowledge-based errors, distractions, excessive workload, and variations in work schedules, were recognized as possible causes. The severity of the MEs was categorized using the NCCMERP index, which classifies MEs from Category A to Category I [19] (Table 1).

**Table 1:** Medication error and risk assessment index according to the NCC MERP.

Category	Description of category
**No error**	
A	Circumstances or events that have the capacity to cause error
**Error, no harm**	
B	An error occurred, but the medication did not reach the patient
C	An error occurred that reached the patient but did not cause patient harm
D	An error occurred that resulted in the need for increased patient monitoring but no patient harm
**Error, harm**	
E	An error occurred that resulted in the need for treatment or intervention and caused temporary patient harm
F	An error occurred that resulted in initial or prolonged hospitalization and caused temporary patient harm
G	An error occurred that resulted in permanent patient harm
H	An error occurred that resulted in a near-death event (e.g., anaphylaxis, cardiac arrest)
**Error, death**	
I	An error occurred that resulted in patient death

### Analysis

Following data collection, the information was inputted into the Statistical Package for the Social Sciences (SPSS) version 23 for evaluation. To determine the significance and potency of the correlation, an odds ratio was utilized along with a 95% confidence interval, in addition to univariate analysis and multiple logistic regression. In the last multivariate model, only the significant predictors of medication errors were included, defined as a p-value of less than 0.05 at a 95% confidence interval.

### Ethical requirements

The study was performed in compliance with ethical principles for human experimentation. As per the National Bioethical Committee of Pakistan, institutional leaders should authorize surveys that do not entail medication administration (National Bioethics Committee Pakistan, 2011). The research was approved by the departmental research committee of the Department of Pharmacy Practice, Faculty of Pharmacy and Health Sciences, University of Balochistan, Quetta. Furthermore, authorization was also obtained from the relevant hospital. The study participants were provided with informed written consent and assured that their personal information would be kept confidential.

## Results

### Inpatient medication file characteristics

The ages of all patients were mentioned in the majority of patient files. Gender was mentioned in every single patient file (100%). The diagnosis was mentioned in most patients (98.8%). (Table 2).

**Table 2:** Demographics of the patients and related information (n = 978).

Characteristics	Group	Patient Followed	Medication Errors (MEs = 274)	Prevalence (%)
**Age (Years)**	18–27 Years	104	47 (45.19%)	4.80
	28–37 Years	134	9 (6.72%)	0.92
	38–47 Years	259	40 (15.44%)	4.09
	48–57 Years	419	147 (35.08%)	15.03
	> 58 Years	62	31 (50.0%)	3.17
**Gender**	Male	679	268 (39.47%)	27.40
	Female	299	6 (2.01%)	0.61
**Hospital**	BINUQ	978	274 (100%)	27.99
**Length of Hospital Stay**	1–5 Days	682	176 (25.80%)	17.99
	6–10 Days	261	79 (30.26%)	8.07
	11–15 Days	35	19 (54.28%)	1.94
**Number of Medicines**	1–3 Medicines	129	29 (22.48%)	2.97
	4–6 Medicine	507	179 (35.31%)	18.30
	More Than 6 Medicines	342	66 (19.30%)	6.75
**Number of antibiotics**	No Antibiotics Prescribed	12	0 (0.0%)	0
	1–3 Antibiotics	962	272 (28.27%)	27.81
	More than 3 antibiotics	4	2 (50%)	0.20

### Inpatient medication file characteristics

#### 1. Demographics of the patients and related information

Out of the 978 patients under observation, 274 medication errors were identified, leading to a prevalence rate of 28.01%. Among the 679 male patients monitored, 268 medication errors were detected, accounting for 39.47% of the total, whereas only six medication errors were identified among the 299 female patients tracked, corresponding to a rate of 2.01%. The age group with the highest prevalence of medication errors was 48–57 years, with a prevalence rate of 15.03%. Most medication errors (18.30% prevalence rate) were noticed among patients taking 4–6 medications. (Table 2).

#### 2. Medication errors: Types, subclassifications, and prevalence

Table 3 showed medication errors, including prescribing, documentation, administration, and dispensing errors. Each type of medication error is further categorized into sub-classifications, such as illegible writing of prescriptions and incomplete filling of prescriptions for prescribing errors. The most common type of medication error is documentation errors, accounting for 43.06% (118) of total medication errors, with a prevalence of 12.06%. Omission errors and extended medication use are the most common sub-classifications of administration errors, accounting for 65.98% (64) and 16.49% (16) of total administration errors, respectively.

**Table 3:** Medication errors: Types, subclassifications, and prevalence.

Type of Medication Error	Number of Medication Errors	Sub Classification of MEs	Prevalence of MEs	Overall Prevalence
Prescribing Errors	34 (12.04%)	Illegible writing of a prescription	2.25	3.47
		Incomplete filling of a prescription	0.72	
		Wrong dose of medication	0.51	
Documentation Errors	118 (43.06%)	Incorrect documentation	6.54	12.06
		Transcription errors	0.82	
		Missed signature	1.84	
		Wrong documentation of dose of medication	2.86	
Administration Errors	97 (35.40%)	Omission errors	6.54	9.91
		Extended use of medication	1.64	
		Wrong frequency of administration	1.12	
		Wrong dose of medication administered	0.61	
Dispensing Errors	25 (9.12%)	Wrong medication	1.33	2.55
		Wrong dosage of medication	1.23	

#### 3. Causes of MEs (n = 274)

Most of these 274 medication errors were determined to have happened in the documentation due to forgetfulness and duty shift, which were significantly associated (p0.05) with MEs. Besides these, lack of attention was significantly associated with prescribing and dispensing errors (p<0.05) (Table 4).

**Table 4:** Causes of MEs (n = 274).

Causes	Type of Errors			
	Prescribing	Documentation	Administration	Dispensing
Duty Shift	15 (5.47)	25 (9.12) *	14 (5.11)	5 (1.82)
Work Overload	4 (1.46)	17 (6.20)	13 (4.74)	5 (1.82)
Forgetfulness	2 (0.73)	25 (9.12) *	15 (5.47)	3 (1.09)
Lack of attention	5 (1.82) **	21 (7.66)	13 (4.74)	4 (1.46) **
Lack of Time	6 (2.19)	12 (4.38)	21 (7.66)	3 (1.09)
Too many prescriptions	2 (0.73)	18 (6.57)	21 (7.66)	5 (1.82)
**Total**	**34 (12.41)**	**118 (43.07)**	**97 (35.40)**	**25 (9.12)**

Significance level:

* <0.05,

** <0.001

#### 4. National coordinating council for medication error reporting and prevention classification of medication errors

Based on the NCC MERP classification of medication errors, the observed errors were divided into categories (Table 5). The majority of medication errors were found to fall under Category A (n = 143), followed by Category B (n = 92), category C (n = 34), and Category D (n = 5).

**Table 5:** National coordinating council for medication error reporting and prevention classification of medication errors.

MEs	Category A	Category B	Category C	Category D	Category E
**Prescribing Errors (n = 34)**	19	6	9	—	—
**Documentation Errors (n = 118)**	85	27	6	—	—
**Administration Errors (n = 97)**	31	50	11	05	—
**Dispensing Errors (n = 25)**	8	9	8	—	—
**Total**	**143**	**92**	**34**	**05**	**—**

#### 5. Risk factors associated with medication errors

Table 6 provides the results of a multiple logistic regression analysis to investigate the factors associated with medication errors. The variables included in the model were age, gender, hospital, length of hospital stay, number of medicines, and number of antibiotics. The most contributing factor associated with medication errors was the number of medicines, with an odds ratio of 9.08 (p < 0.001) for patients receiving 4–6 medicines and an odds ratio of 4.23 (p < 0.001) for patients receiving more than 6 medicines, both compared to patients receiving 1–3 medicines. Age was also significantly associated with medication errors, with patients aged 38–47 years having an odds ratio of 2.72 (p = 0.009) and patients aged >58 years having an odds ratio of 2.10 (p = 0.619), compared to patients aged 18–27 years. Gender was also a significant factor, with female patients having significantly higher odds of medication errors than male patients, with an odds ratio of 26.62 (p < 0.001).

**Table 6:** Regression analysis of factors associated with medication errors.

Variable	Coefficient (β)	Standard Error	t-value	p-value
**Age (reference: 18–27 years)**				
**28–37 years**	-1.61	2.67	-0.60	0.548
**38–47 years**	0.41	1.51	0.27	0.785
**48–57 years**	2.72*	1.04	2.62*	0.009
**> 58 years**	1.05	2.10	0.50	0.619
**Gender (reference: Male)**				
**Female**	-26.62**	6.55	-4.06**	<0.001
**Hospital (reference: BINUQ)**				
**Other Hospitals**	-10.64	7.64	-1.39	0.163
**Length of Hospital Stay (reference: 1–5 Days)**				
**6–10 Days**	0.45	1.52	2.65	0.456
**11–15 Days**	0.10*	0.03	3.28**	0.001
**Number of Medicines (reference: 1–3 medicines) 4–6 medicines**	9.08**	2.15	4.23**	<0.001
**> 6 medicines**	2.64	2.23	1.18	0.240
**Number of Antibiotics (reference: No antibiotics) 1–3 antibiotics**	14.28**	3.64	3.92**	<0.001
**> 3 antibiotics**	3.41	21.49	0.16	0.874

p<0.05,

** p<0.01

## Discussion

The evaluation of medication errors in Quetta, Pakistan, among patients with kidney diseases identified several significant factors associated with medication errors. Specifically, patients with longer hospital stays of 6–10 days had higher odds of medication errors (OR = 1.52, p = 0.456). Patients receiving 1–3 antibiotics had significantly higher odds of medication errors (OR = 14.28, p<0.001). These findings suggest that healthcare providers should focus on interventions to reduce medication errors in patients with longer hospital stays, those receiving antibiotics, those taking multiple medications, and patients in specific age and gender groups.

According to the findings of this study, MEs account for 27.99% of medication errors that occur in hospitals. This result was higher when compared to studies conducted in West Ethiopia and India, which revealed medication errors in both countries at 46% and 42.85%, respectively [26, 27]. We discovered that 27.99% of the patients in this study had come into contact with at least one ME. Some strategies that could be used to lower the high rate of medication errors include the creation and implementation of a computerized physician order entry (CPOE) system, ward-based clinical pharmacists, the avoidance of verbal orders, the reading back of verbal orders, medication reconciliation, double checking, and patient (parent) active participation in care [28].

The burden of medication errors on the healthcare system is rising. The risk factors for medication errors were similar to those from prior research; older age, a burdened healthcare system, more prescription medicines, and comorbidities were all strongly linked to an increased risk [29, 30]. However, this outcome is three times greater than that of the American trial, in which 5.7% of patients had three or more mistakes and 28.6% had at least one ME [12]. This variation may result from variations in hospital settings, including variations in the training levels of healthcare professionals, the accessibility of support systems, the makeup of the healthcare team, variations in data collection techniques, and variations in the definitions and interpretations of errors. The high rate of medication administration errors observed may be attributed to various factors, including professional factors [31, 32].

The current study shows that most errors were incorrect documentation and omission errors, with a prevalence of 6.54, respectively because of the hospital Failures by a doctor to order a vital medication that a patient is taking, by a nurse to give medication as directed, or by a pharmacy to fill a prescription. The results agreed with those of other studies. According to Acheampong et al., there were more omission errors in their study, with a prevalence of 77.6% [33]. When switching from one shift to another, omission errors may occur because the Nurse failed to record the medication process on the treatment sheet. Due to this, some of the prescribed drugs were not given. The least frequent drug error reported in this investigation was dose error. The prevalence was relatively lower than other emergency rooms’ data [34].

The current study showed that the Illegible prescription writing has been associated with medication errors in hospital settings. Illegible handwriting can cause treatment delays, unnecessary tests, and inadequate dosages, which can cause discomfort and even death. The results were in line with Another reason for prescription errors: handwriting that was either illegible or not legible [35]. The pharmacist will have less difficulty filling prescriptions if the patient’s age, sex, and diagnosis are included along with the medication’s generic name. However, without such information, he must ask the prescriber for clarification more frequently. The doctor is legally obligated to write legible prescriptions and is also accountable for any incorrect prescription interpretations made by the pharmacist [36]. The current study’s findings are also comparable with Knudsen et al. (2007) analyzed self-reporting in community pharmacies and discovered a strong link between dispensing errors and illegible handwriting [37].

The current study showed that lack of attention was significantly associated with prescribing and dispensing errors. This finding was comparable: lack of focus, inadequate training, and the absence of guidelines for safe drug administration practice were also strongly associated with medication administration errors. This finding is supported by the World Health Organization’s harm-free drug strategies released in 2017. The WHO will develop measures to promote patient safety and decrease pharmaceutical errors by as much as half in the five years from 2017 to 2022 [38].

Medication errors can also be decreased by using computers to create prescriptions. A computerized system can be tailored and integrated with patient information, such as physiological characteristics, known allergies, and current drug availability information, and designed to deliver the finest possible care. Such an integrated system can also give drug-specific information, warnings about potential overdose, drug interactions, and other things before a prescription is finished. For patients receiving long-term treatment, computerized prescribing can increase the frequency of monthly prescriptions in addition to technical input. Once this prescription has been written, it can be electronically transmitted to the dispensary so that distribution packets can be made before the patient shows up, cutting down on waiting time. The development of this technology, its implementation in several hospitals, and worker training continue to be the key challenges in this endeavor. The key hurdles to ensuring the regular usage of this technology will be updating the database in light of new evidence, securing data, maintaining secrecy, and routine troubleshooting.

### Limitation

This study has several restrictions. The study’s main drawback is that it was conducted at just one location. The public, private, and semi-private wards were all off-limits. A medication error that occurred at night went unreported.

### Future recommendations

Based on our study findings, we recommend implementing several measures to reduce medication errors in patients with kidney diseases. Healthcare providers should receive training on proper documentation practices to minimize documentation errors. Healthcare providers should also be encouraged to pay close attention to the prescribing and dispensing processes to prevent inattention-related errors. Limiting the number of medications prescribed to patients, especially those with kidney diseases, may also help reduce the risk of medication errors. Additionally, healthcare facilities should consider implementing medication reconciliation programs to ensure that patients receive the correct medications at the correct dosages. Future research should focus on the effectiveness of these measures in reducing medication errors and improving patient outcomes.

## Conclusion

In conclusion, the study highlights the significant issue of medication errors in patients with kidney diseases in Quetta, Pakistan, with a prevalence of 27.99%. Several contributing factors were identified, including longer hospital stays, receiving multiple medications and antibiotics, illegible prescriptions, lack of attention, incorrect documentation, and omission errors. To prevent medication errors, healthcare professionals should take necessary precautions such as clear documentation and attention to detail. Healthcare organizations should also provide training and resources to improve patient safety. The conventional handwritten prescription process is prone to errors, particularly in transcription, due to illegible handwriting. Implementing standardized medication administration protocols, providing regular training, promoting a culture of safety and reporting, regularly reviewing medication policies, and utilizing technology such as electronic prescribing systems and medication barcoding can help reduce medication errors in older adults.

## Supporting information

S1 File (SAV)
